# HANDS-ON: Training Simulation in Surgery

**DOI:** 10.1590/1516-3180.2022.1413230223

**Published:** 2023-04-07

**Authors:** Carlos Manuel de Almeida Brandão, Paulo Manuel Pêgo-Fernandes

**Affiliations:** I MD, PhD. Attending Physician Cardiovascular Surgery Department, Heart Institute, University of Sao Paulo Medical School, São Paulo, SP, Brazil; II MD, PhD. Vice Director, Faculdade de Medicina FMUSP, Universidade de Sao Paulo, Sao Paulo, SP, BR; Full Professor, Department of Cardiopulmonology, Instituto do Coração, Hospital das Clinicas HCFMUSP, Faculdade de Medicina, Universidade de Sao Paulo, Sao Paulo, SP, BR; Director, Scientific Department, Associação Paulista de Medicina (APM), São Paulo (SP), Brazil.

Methods of the teaching-learning binomial in surgery has undergone major transformations in the last decade, moving from purely informational content models of environments that stimulate theoretical “know-how” by incorporating skills and competencies. There has been a paradigm shift in the training of surgical residents from the traditional ‘‘apprenticeship’’ model to a hybrid one, involving simulation training. This shift has been in response to several current challenges in residency training, including time constraints, patient safety concerns, financial costs, and decreasing durations of training programs.^1^ During the past several years, major shifts in surgical education have brought into question whether surgical residency programs could truly ensure competency and technical proficiency in these surgeons.^2^

Simulation is an increasingly vital component of graduate medical education and becoming the standard of practice in many residency programs, particularly in surgical specialties, owing to the need for moving basic skill acquisition out of operating rooms into surgical skill laboratories. Deliberate practice is an educational technique that aims to improve performance through intense training and preparation. These steps include repetition, assessment, and feedback, which lead to performance improvement.^3^

Since a wave of innovation using new technologies has introduced a series of procedures with less invasive potential, such as minimally invasive surgery and robotics, training in surgery has become progressively more complex, occupying an important space in surgical treatment. Residents and young surgeons must learn these new techniques in a safe and effective way. Therefore, the advent of several simulators in surgery to practice activities is called “Hands-On.”

The “Hands-On” is a different strategy for teaching and learning because it allows for interaction between experts (surgeons with recognized expertise and competence) with surgeons on different learning curve phases including residents, junior surgeons or even those with several years of surgical practice. This strategy differs from other teaching activities once trainees are directly involved in the whole procedure by “using their hands.” Trainee is incentivized to observe all steps of operations, perform tactical movements and actions ordered in a logical sequence, or may incorporate new skills. All this happens without the urgency of time and, importantly, under qualified and committed guidance and supervision of experts.

At the same time, it promotes teaching and allows for both correction and evaluation of performance-inducing satisfaction in trainees by creating a pleasant sense of mastery of new skills or consolidation of previous ones that could now be executed with a more refined technique. These new skills are incorporated into the salutary practice of exhaustive repetition, which will automate them. After their final registration in the brain centers that integrate knowledge with motor skills, these new skills emerge automatically when requested, completing the cycle of see-search-understand-perform.^4^

It is also challenging for institutions responsible for training programs because they are expected to offer an efficient and adequate curriculum, which relies on the paradox of providing safe surgical opportunities without compromising postoperative outcomes and excellent patient care. Operating room surgical training has significant limitations: it provides a short time to develop technical skills and has low tolerance for learning mistakes.

Regarding traditional simulation models for surgery training, despite their high fidelity, human cadaver training has been discouraged for two main reasons. First, the corpses are not always available for use under good conditions for preservation. The second reason might be the short period of time spared for training, since those patients have to be handed out to their families for ceremonials.

Conducting training procedures on animal models offers the closest scenario to surgery in humans. However, completion of this kind of training requires sacrificing these animals, culminating in great opposition by animal protection organizations as well as by the general population. This traditional method has well-known limitations, such as the need for a broad framework for hosting, maintaining, and preparing these animals and their subsequent disposal, which, in addition to high costs, also requires involvement of many professionals for the correct execution of these tasks. These facilities are routinely available only in medical schools, to which undergraduate or graduate students usually have restricted access.

Biological models, compared with animal models “*in vivo”*, have the advantage of storage capacity for days in the refrigerator, and lower costs, which is very advantageous considering the current financial constraints of most residency training programs. In addition, they enable realistic tissue sensations and excellent anatomical correspondence. In this context, we suggest that incorporating the simulation of biological models in our residency program could improve surgical training and patient safety owing to its potential to avoid surgical technical failure that would compromise the outcomes, bringing more safety, efficacy, and better long-term results.

Recognizing these challenges about technical skills acquisition for cardiovascular surgery, we started our simulation training program. This program was on biological models for the medical residency of cardiovascular surgery at our institution, the “Hands-on” course with biological models in 2015, for cardiovascular surgery residents in the Heart Institute of University of São Paulo Medical School. A practical evaluation of the performance of specific skills was conducted at the end of the training program. The high scores obtained by residents revealed the efficiency of the simulation in the acquisition of skills. In addition, the residents’ responses to the subjective analysis revealed high satisfaction with the training program. They highlighted realistic tissue feel and anatomical representation as positive aspects, as well as the possibility of performing a specific technique multiple times, thereby improving skill acquisition. In general, most of the residents reported that the program could provide a realistic experience and be valuable in teaching surgical skills.^5^ We observed that biological models with porcine or bovine hearts provided a high degree of realism. In the case of cardiac surgery, where surgical failure may result in patient morbidity and mortality, such a realistic simulation could reproduce better surgical results, which is essential before clinical application.

The incorporation of new training techniques, such as “Hands-on” simulators, allows for improvements in resident preparedness, training of new technologies by practice surgeons, minimizing possible technique failures, and allowing for better efficacy in the procedures and better results. These moments represent a unique opportunity for a desirable interaction, in that the more experienced ones could help qualify this critical mass of professionals eager to attain new knowledge but also willing to incorporate specific technical skills as basic support for their professional performance.^6^

In the cognitive domain, the transmission and retention of essential theoretical knowledge are required and of paramount importance for the judgment and proper handling of each patient. This part has been widely covered by many mini-courses offered at numerous conferences in different areas of the specialty as well as continuing medical education programs carried out by schools and educational institutes created and maintained by specialty societies and supported by pharmaceutical companies for equipment and instruments in surgery.^7^

The congresses of some specialties are modified each year to discuss the incorporation of new technologies by presenting the results of numerous well-designed studies conducted to validate, disseminate, and extend their use in daily practice. Watching an operation, usually a complex case or new procedure, performed at distance in a specialized center and broadcasted to an audience, offers the participating surgeons an opportunity to interact with the team that performs surgery and learn useful surgical aspects. However, this does not endow observers with new skills. In addition, this format has certain limitations, including legal issues. The surgical team responsible for surgery was subjected to stress above the usual levels. They could not repeat the tactical maneuvers performed because of the imperative need for continuing the operation. Unclear steps or details, even if explained well, cannot be repeated. The operation needs to have a normal course and patient must not be subjected to additional risks, such as stopping at each step of the procedure, to allow for controversial debates or opinions. Traditional video sessions, in which procedures can have their technical details presented and discussed, allowing for pauses or repetitions when necessary, are instructive in providing opportunities for learning technical and tactical details without putting pressure on the surgical team without subjecting patients to additional risks. This mode helps understand how to overcome difficulties in their implementation and new ways of executing it. Although it is a useful and attractive format, it does not provide new psychomotor skills to those attending the activity. Therefore, creative and innovative ways of transmitting knowledge, as described above, do not directly involve a community of observers in theatres for surgical procedures. Observers can even assimilate the steps and various tactical maneuvers essential to the operation. However, these do not provide them with the ability to implement them.

The task of producing scientific knowledge and validating it through the current methods of evidence-based medicine belongs primarily to universities. Through their institutes and research laboratories, universities are prepared for this crucial stage in developing medical science. On the other hand, the medical specialty societies must be in charge for the task of training and retraining the graduates of these educational institutions who completed their residency program or fellowship in surgery to maintain excellence in their daily practice.

Some studies have validated the need for simulation in surgery.^8^ Surgical simulation allows medical residents to perform surgeries in a less stressful environment and may provide structured graduate training for technical skills. Furthermore, educators recognize this activity as a method by which expertise may be developed and assessed. Another positive aspect of simulation-based skills training in surgery is that educators can spend more time teaching in less stressful environments, promoting a better understanding and retention of skills.

Whether the improved performance in the simulation laboratory is transferable to the operating room is not easily addressed. However, most trainees reported that they felt much better prepared and less anxious about performing each skill in an operating room environment. These findings support those in the published literature that structured teaching sessions improve learners’ confidence level.^9^

In addition, concerns have been raised regarding the safety of training surgeons when performing surgeries. In fact, there is a growing demand for improved clinical results and outcomes, with intense public scrutiny of their clinical performance. That is why surgical training in this field requires balance between standard of care delivered to patients and provision of sufficient operative exposure to trainees who are the cardiac surgeons of the future.

The perception that a procedure requires fellowship training is a possible explanation why most core and advanced surgical skills are considered above residency training. Proving competency in a controllable environment may allow residents to gain autonomy in operating rooms. Faculty surgeons may be more at ease because residents have demonstrated their knowledge and skills before entering the operating room. A study conducted in general surgery found that 80% of faculty members did not expect graduating residents to perform complex operations.^10^

There is a pressing need to incorporate simulation-based training into existing and future surgical residency programs. Since mandates for quality measures and shorter training periods emerge, teaching alone, using the traditional ‘‘apprenticeship’’ model in the operating room, is no longer sufficient. High-fidelity, low-technology tools such as fresh tissue cadaver laboratories and virtual operating rooms might be important adjuncts to successful curriculum implementation. Each program should recognize that a needs assessment can help focus on curricular content and that implementation will vary depending on resources, faculty time, and available educational time. These programs could benefit from defining the specific needs that are important for the widespread and local implementation of simulation-based training in surgery.

The combination of patient safety concerns, changes in resident education, and more complex procedures for high-risk patients has generated a greater interest in simulation-based learning in training. Considering the current educational environment and importance of training the next generation of surgeons, simulations represent a feasible alternative. We strongly suggest that simulation may contribute to the development of technical skills and procedural knowledge required for adequate performance in the operating room.
